# CRISPR-Cas9-Based Technology and Its Relevance to Gene Editing in Parkinson’s Disease

**DOI:** 10.3390/pharmaceutics14061252

**Published:** 2022-06-13

**Authors:** Mujeeb ur Rahman, Muhammad Bilal, Junaid Ali Shah, Ajeet Kaushik, Pierre-Louis Teissedre, Małgorzata Kujawska

**Affiliations:** 1 Department of Toxicology, Faculty of Pharmacy, Poznan University of Medical Sciences, Dojazd 30, 60-631 Poznan, Poland; mujeeb.rahman@student.ump.edu.pl; 2 College of Biotechnology, Tianjin University of Science and Technology, Tianjin 300457, China; bilalofasia@gmail.com; 3 College of Life Sciences, Jilin University, Changchun 130012, China; junaid1316@mails.jlu.edu.cn; 4 Fergana Medical Institute of Public Health Uzbekistan, Fergana 150110, Uzbekistan; 5 NanoBioTech Laboratory, Health System Engineering, Department of Environmental Engineering, Florida Polytechnic University, Lakeland, FL 33805, USA; akaushik@floridapoly.edu; 6 School of Engineering, University of Petroleum and Energy Studies (UPES), Dehradun 248007, Uttarakhand, India; 7 Institut des Sciences de la Vigne et du Vin, Université de Bordeaux, EA 4577, Œnologie, 210 Chemin de Leysotte, F-33140 Villenave d’Ornon, France; pierre-louis.teissedre@u-bordeaux.fr; 8 Institut des Sciences de la Vigne et du Vin, INRA, USC 1366 INRA, IPB, 210 Chemin de Leysotte, F-33140 Villenave d’Ornon, France

**Keywords:** Parkinson’s, CRISPR-Cas9, gene therapy, delivery, applications

## Abstract

Parkinson’s disease (PD) and other chronic and debilitating neurodegenerative diseases (NDs) impose a substantial medical, emotional, and financial burden on individuals and society. The origin of PD is unknown due to a complex combination of hereditary and environmental risk factors. However, over the last several decades, a significant amount of available data from clinical and experimental studies has implicated neuroinflammation, oxidative stress, dysregulated protein degradation, and mitochondrial dysfunction as the primary causes of PD neurodegeneration. The new gene-editing techniques hold great promise for research and therapy of NDs, such as PD, for which there are currently no effective disease-modifying treatments. As a result, gene therapy may offer new treatment options, transforming our ability to treat this disease. We present a detailed overview of novel gene-editing delivery vehicles, which is essential for their successful implementation in both cutting-edge research and prospective therapeutics. Moreover, we review the most recent advancements in CRISPR-based applications and gene therapies for a better understanding of treating PD. We explore the benefits and drawbacks of using them for a range of gene-editing applications in the brain, emphasizing some fascinating possibilities.

## 1. Introduction

Neurodegenerative diseases (NDs) are conditions characterized by the progressive loss of neurons in the brain and peripheral nervous system and the deposition of proteins with altered physicochemical properties. Such proteins are used to classify NDs at the molecular level. β-Amyloid, α-synuclein, huntingtin protein, prion protein, tau, TAR-DNA-binding protein 43 kDa, and fused-in sarcoma protein are the most common proteins that contribute to Alzheimer’s disease (AD), Parkinson’s disease (PD), Huntington’s disease (HD), transmissible spongiform encephalopathies, tauopathies, and amyotrophic lateral sclerosis (ALS), respectively [1,2,3,4,5]. The diseases characterized by the delayed appearance of symptoms and degeneration in the brain include AD, PD, HD, and others, which predominantly affect everyday activities [6]. Several mutations in the genes encoding for α-synuclein and PINK1 in PD, amyloid precursor proteins, presenilin, and tau in AD, and expanded CAG repeats in HD are known to contribute to the development of age-related neurodegeneration [7,8,9]. Signifying a state in which neurons are gradually lost, neurodegeneration affects a person’s cognitive behavior, increasing their reliance on others over time [10]. Besides genetic factors, many environmental ones are linked to an increased risk of NDs [11]. PD is the most rapidly developing neurological condition, affecting up to 2% of people over 60 [12]. The molecular processes that underpin the pathophysiology of sporadic PD are still a mystery. As a result, causative therapies remain elusive [13]. However, the degradation of the dopaminergic neurons (DNs) in the nigrostriatal pathway is a primary cause of chronic and increasing motor impairment; PD is now recognized as a systemic disorder affecting various nervous system regions [14]. Most cases of PD occur in a sporadic form [15]. The diagnosis of PD may be hard to confirm completely because brain autopsy remains the most well-established and conclusive method. Hence, it is necessary to understand the disease’s distinct characteristics and manifestations in order to distinguish actual PD from other related disorders [16].

There are few, if any, therapy options available for most hereditary diseases [17]. As a result, gene-editing tools such as transcription activator-like effector nucleases (TALENs), zinc finger nucleases (ZFNs), and meganucleases, as well as CRISPR (clustered regulatory interspaced short palindromic repeats)-Cas9 (CRISPR-associated enzyme), have sparked a significant interest. These technologies can edit, replace, and change defective sites on the genome to treat a particular neurodegenerative disorder (PD, AD, and HD). Using these technologies to introduce normal genes into the damaged portion of the genome may stop disease progression. However, there are still challenges in correctly excising only the defective areas of the gene. CRISPR-Cas9 appears to be the most promising gene-editing technique available because of its ease of use, efficacy, cost-effectiveness, and capacity to edit several genes at once [18,19]. The Nobel Prize in Chemistry was recently awarded to Emmanuelle Charpentier and Jennifer Doudna for their work on CRISPR-Cas9, a method for editing DNA. The Nobel Committee honored the two scientists for their discovery that a microbial immune system can be turned into a tool for editing genomes with high precision simply and inexpensively [20]. This review article discusses the CRISPR-Cas9-based technology and its perspective for application in PD.

## 2. CRISPR-Cas

### 2.1. History

In 1987, bacteria were found to insert 32-nt (nucleotide) spacer sequences into 29-nt repeat sequences in CRISPR loci whenever they came into contact with phage DNA, leading to the discovery of the CRISPR-Cas system [21]. Similar repeating sequences were discovered in other *E. coli* strains: enterobacteria closely related to E. coli, and Shigella dysentery in the following years [22]. In 1993, Mojica and colleagues found the CRISPR repetitive sequence in archaea while researching the effects of salinity on the growth of Haloferax mediterranei. Although there was no similarity between these sequences and E.coli repeats, these researchers discovered a lengthy DNA sequence in the genome of these archaea that consisted of regulatory repeats [23]. In the CRISPR-Cas era, 2005 is regarded as a pivotal year because it was recognized that the spacer sequences were derived from phage genomes [24]. Together with the finding that Cas-gene encoded proteins with putative helicase and nuclease domains [25,26,27], and that CRISPR loci can be transcribed [28], It was recommended that CRISPR-cas is an adaptive system that may use antisense RNAs as a memory marker of past invasions [29]. In 2007, it was suggested that the CRISPR system could be used as an adaptive immune defense for bacteria and archaea against phage attacks. For example, adding or deleting spacer DNA homologous to phage DNA can alter the resistance of Streptococcus thermophilus to phage invasion [30]. In 2008, mature CRISPR RNAs (crRNAs) were determined to act as guides in a complex with Cas proteins in E. coli, preventing viral replication [31]. The CRISPR-Cas system’s DNA targeting activity was identified in the pathogen Staphylococcus epidermidis the same year. For nearly 20 years after their discovery, the function of these repeats remained unknown. Multiple direct repeats (DRs), short regulatory spaced repeats, and large clusters of tandem repeats have all been proposed as names for these repeats. Jansen and coworkers invented the word CRISPR, which has now gained acceptance among researchers since it reflects the structural properties of repeats [32,33,34,35].

### 2.2. CRISPR-Cas System

The classification of the CRISPR-Cas system is very challenging because there are no universal Cas proteins that could have served as phylogenetic markers. Consequently, the classification is based on many features, including the layout of Cas operons, signature Cas genes, and phylogenies of conserved Cas proteins [36]. There are two classes (Class 1 and Class 2), six types (I–VI), and 33 subtypes of CRISPR-Cas, according to a classification published in 2020 [37]. Multi-subunit effector complexes are seen in Class 1, while single protein effector modules are found in Class 2. Identifying two new types and several subtypes of the Class 2 CRISPR-Cas system resulted in more research and analysis of the system. The type VI systems, out of the two recently identified and defined CRISPR types, were the only ones that targeted RNA. In some circumstances, the class 2 systems have a unique feature in which the effector protein is also involved in processing pre-crRNA (CRISPR RNA) [38]. The CRISPR-Cas system’s two major classes, 1 and 2, have a solid basis of variation. The multi-subunit crRNA effector complex is classified as Class 1, while the single crRNA effector complex has been classified as Class 2. The Class 1 CRISPR-Cas system has been subdivided into types (I, III, and IV) and further into subtypes. Similarly, Class 2 is divided into three types: II, V, and VI, each further classified into multiple subtypes. The most widely used CRISP-Cas system is the type II CRISPR-Cas system which has been obtained from Streptococcus pyogens (SpCas9) [39,40]. The two main components of the CRISPR-Cas9 system are single guided RNA (sgRNA) and RNA guided Cas9 endonuclease [41]. There are two nuclease domains of Cas9, named RuvC and HNH, each breaking a single strand of targeted double-stranded DNA [42]. The RuvC domain cleaves the non-complimentary strand of dsDNA interacting with crRNA, while the HNH domain cuts the complementary strand [43]. A single-guide RNA (sgRNA) is a condensed form of crRNA and tracrRNA [44]. The Cas9 nuclease and sgRNA combine to form a Cas9 ribonucleoprotein (RNP) that can bind to and cleave the specific target in DNA [43].

Furthermore, the desired task of CRISPR-Cas9 systems is provided by the protospacer adjacent motif (PAM), which is an area inside an invading DNA that helps bacteria in differentiating pathogenic genetic information from its own [45,46]. If the spacer sequence is entirely identical to PAM, the CRISPR-Cas9 system will exclusively target plasmid or viral genetic materials by generating double-stranded (ds) DNA breaks in the invaded DNA [47]. As a result of these findings, researchers have determined that the CRISPR-Cas9 system can be employed as a new genome-editing tool in various organisms. It causes double-strand breaks (DSB), which can be fixed by either the homologous directed repair pathway (HDR) or error-prone non-homologous end junction (NHEJ) pathway, which are both endogenous self-healing processes [48]. NHEJ is more effective than HDR in most cases because it does not depend on a nearby homology donor and is also active for approximately 90% of the cell cycle [49]. NHEJ can integrate random insertion or deletion (indel) into the cleavage site, resulting in frameshift mutation or early termination codon in the open reading frame of the target gene so as to inactivate it [50,51]. However, HDR can introduce precise genomic changes at the target site using homologous DNA repair templates [52,53]. In addition, many sgRNAs targeting one or more genes can be used to create large deletions and knock out many genes at the same time [54,55].

## 3. Parkinson’s Disease

Movement disorders, such as PD and HD, are some of the most frequent NDs. They are classified as complex neurological diseases and are characterized by affected body movements [56]. PD is a heterogeneous neurodegenerative condition that affects an estimated 10 million people globally [57]. The progressive loss of DNs in the substantia nigra pars compacta (SNpc) causes motor symptoms such as rest tremors, bradykinesia, and rigidity, which constitute the core of PD clinical characteristics [58]. This neuronal loss is followed by the appearance of cytoplasmic inclusions of Lewy bodies (LBs), which are primarily made of aggregates of misfolded α-synuclein protein and may spread in a prion-like way between synaptically interconnected areas [59]. In vivo, in vitro, and autopsy studies support that α-synuclein spreads in a prion-like manner [60].

In addition, non-motor symptoms like cognitive decline, sleeping problems, depression, intestinal dysfunction, and anxiety are also becoming more commonly recognized as key factors in a patient’s standard of living and impairment [61]. PD prevalence rises with age (from 40–49 years up to people aged >80 years), and it is gender-dependent, with it being twice as common in males than in females [62,63]. The incidence rate of PD worldwide is increasing, and by 2040, the number of people suffering from the disease is expected to be close to 12 million, prompting some scholars to list it as a pandemic [64,65]. The majority of PD patients are classed as idiopathic, with approximately 10% having a proven monogenic cause (familial PD). Idiopathic PD’s etiology is unknown, but genetics, aging and environmental factors and their interactions have a role in the disease’s onset and development. Ninety common polymorphisms linked to the development of PD have been discovered in genome-wide studies [11,66], and the influence of genetic factors on the clinical heterogeneity and development of PD is still being investigated. Currently, the most common treatment for PD is symptomatic medication therapy. No mechanism-based treatment methods to prevent, regulate, or minimize the clinical signs of PD have been developed [67,68]. Additionally, the symptomatic therapeutic modalities used have many side effects. With the progression of the disease, the nonlinear pharmacodynamics of dopamine (DA) replacement therapy complicates the optimization of a treatment regimen [69]. A few cell replacement therapy researchers have demonstrated the feasibility of producing DNs from human embryonic stem cells (hESCs) and implanting these cells in animal PD models [70,71]. The early findings revealed that DA levels in the brains of experimental animals had increased [72]. However, this technique has several unresolved issues, including the possibility of immunologic response, brain tumors, ethical considerations, phenotype instability of hESC-derived DA neurons, and the need to assess the treatment’s effectiveness and safety in PD patients.

## 4. Application of CRISPR-Cas in PD

Based on the potential pathogenic function of microglia and α-synuclein’s demonstrated ability to destroy aberrant intracellular α -synuclein filaments and prevent DA neuron damage [73,74], vaccines against α-synuclein might be an effective treatment option. However, no research focused on this method has yet been published. New mechanistic studies are needed to better understand the pathogenesis of PD, in which environmental and genetic variables contribute to a range of aberrant metabolic pathways and incorrect interactions between different macromolecules. The CRISPR-Cas9 system—a revolutionary technology created in the last decade that allows for immediate and accurate genome editing in nearly any living species—seems to be a promising approach in PD also [75,76]. CRISPR-Cas9 offers the possibility to accelerate basic research, focusing on elucidating the pathogenicity of neurological diseases and leading to new therapies, according to several recent articles, mainly for PD [77,78]. CRISPR-Cas9 technology is more succinct, versatile, and cost-effective than other gene-editing methods, resulting in its increasing popularity [41]. The CRISPR-Cas9 system enables us to edit candidate genes (Table 1) to generate appropriate animal and cell line models, significantly improving our understanding of the disease. In the future, it may become an important tool for effective and valuable gene therapy, which is considered to be a new therapeutic strategy for PD [79].

**Figure 1:**
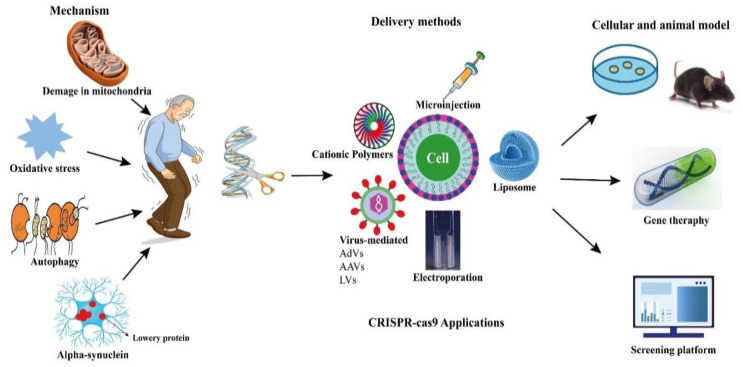
Potential applications of CRISPR-Cas9 in PD.

CRISPR-Cas9 technologies have been proposed to offer a number of genomic modifications in addition to site-directed gene editing. CRISPR interference (CRISPRi) and CRISPR activation (CRISPRa) technologies have also been used to regulate the expression of target genes by making precise base modifications with a catalytically dead nuclease (dCas9) [102,103,104,105]. In addition, they have been adapted as tools for gene location detection [106], epigenetic research [107], and even modified RNA targeting (Figure 1) [108].

## 5. Gene Therapy and PD

Gene therapy was first introduced in 1972 as a method of replacing defective DNA with “good” DNA that may be used to treat disorders at the DNA level [109].

Neuropathological findings show a link between mutations in the α-synuclein (SNCA) gene and the severity of neuronal degeneration in the SN region of PD patients. Therefore a considerable effort is made to edit this target. Kantor and colleagues focused on the development of an epigenetic-based therapeutic approach targeting SNCA expression regulation. As SNCA transcription is regulated by DNA methylation at SNCA intron 1, a level in the brain that differs between PD patients and controls, they created a technique for targeted DNA methylation editing within intron 1 using an all-in-one lentiviral vector. The system was made up of CRISPR-deactivated Cas9 (dCas9) coupled to the DNA-methyltransferase 3A catalytic domain (DNMT3A). Applying the system, they downregulated SNCA mRNA and protein in human induced pluripotent stem cell (hiPSC)-derived DNs from a PD patient with the triplication of the SNCA locus. PD-related cellular phenotype characterized by, for example, mitochondrial ROS production and cellular loss were rescued by the guide RNA (gRNA)-dCas9-DMNT3A systems. Moreover, the fine-tuned downregulation of SNCA level with the CRISPR-dCas9 tool was suggested to be used for a novel epigenetic-based therapeutic approach against PD [77]. Hyung Ho Yoon et al. studied the CRISPR-Cas9 tool in vitro and in vivo to eliminate A53T-SNCA. In vitro, an AAVS comprising the single guided RNA and SaCas9-KKH targeting A53T-SNCA greatly decreased the expression of A53T-SNCA. Moreover, they examined the therapeutic effects of this approach in the viral A53T-SNCA overexpressing rat model of PD. Overexpression of α-synuclein, motor symptoms, dopaminergic neurodegeneration, and reactive microgliosis was reduced dramatically when the A53T-SNCA gene was deleted. The findings support the use of the CRISPR-Cas9 technique to minimize A53T-SNCA-specific PD [110]. Furthermore, Y Chen et al. used the CRISPR-Cas9n strategy to establish SNCA−/− and SNCA+/− cell lines by deleting the endogenous SNCA gene, which encodes for α-synuclein, in a clinical-grade hESC line. As cell replacement in PD patients has been demonstrated to be susceptible to the host-to-graft transfer of α-synuclein pathology, the developed hESC lines converted into mDA neurons were challenged with synthetic α-synuclein fibrils. The recombinant neurons showed significant resistance to Lewy pathology, supporting the use of CRISPR/Cas9n-mediated in removing SNCA alleles against PD [111].

Inoue et al. have demonstrated that manipulating the expression of a novel 13-kDa protein (p13) inducing mitochondrial dysfunction and related apoptosis may be a promising therapeutic intervention in PD. In p13-deficient mice generated by using the CRISPR/Cas9 method, there was no motor dysfunction or DAergic neuron destruction following treatment with model neurotoxin MPTP. Moreover, they demonstrated that p13 knockout prevented MPTP-induced impairment of complex I assembly in the midbrain of mice [112].

The therapies developed for targeting PD can be disease-modifying and non-disease-modifying. Platelet-Derived Growth Factor (PDGF), Glial Cell Line-Derived Neurotrophic Factor (GDNF), Brain-Derived Neurotrophic Factor (BDNF), and Neurturin are several disease-modifying targets that can decrease the development of PD. While non-disease-modifying Vascular Endothelial Growth Factor A (VEGF-A) and Cerebral Dopamine Neurotrophic Factor (CDNF) are symptomatic, they do target GABA (Gamma-aminobutyric acid) or dopamine synthesis [113,114].

Gene editing has the potential to uncover the molecular basis of PD, find new therapeutic targets, and eventually generate new gene treatments. Upregulation and downregulation of gene expression or selective editing of key genes known to be modified in PD, such as PRKN, GDNF, PINK1, and AADC (aromatic L-amino acid decarboxylase), can be used to correct defects in the molecular pathways related to PD [113]. Gene editing could still be a viable technique for restoring the activity of important biological pathways that have been interrupted and may be contributing to PD.

**Figure 2:**
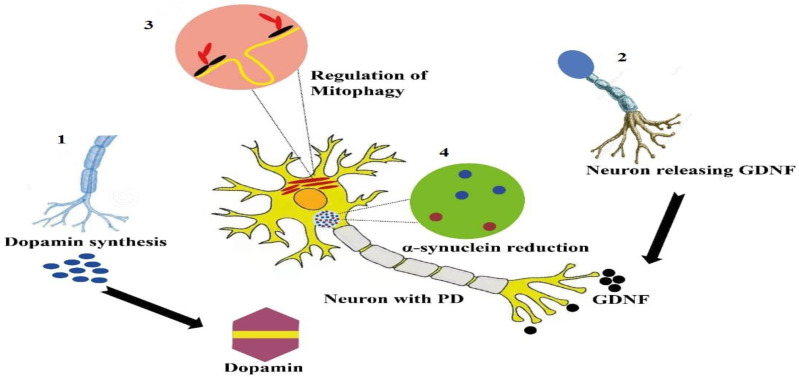
Four categories of gene-editing strategies for PD based on the therapeutic target are (1) enhancement of dopamine synthesis, (2) increase in the availability of trophic factors and neuromodulation, (3) activation of mitophagy, and (4) α-synuclein clearance in the brain.

Gene editing is a viable approach for restoring the function of essential biological pathways that have been disrupted and cause PD symptoms. Based on therapeutic goals in PD, four categories of this approach are being developed [115]. The first strategy is to boost brain DA bioavailability. In order to stimulate brain regeneration, the second technique relies on neurotrophic factors and neuromodulation in the subthalamic nucleus (STN). A third strategy focuses on genes involved in mitochondrial pathway and mitophagy. Lastly, the fourth technique involves decreasing α-synuclein synthesis, which helps to alleviate the effects of modified mitochondrial pathways (Figure 2) [116,117,118].

These techniques aim to change the mitochondrial, autophagic, and lysosomal metabolic pathways, which have been linked to PD and neuron survival. In gene-editing studies of PD, the DA pathway and neurotrophic factors have received the most attention. Neurotrophic factors can be manipulated to reduce symptoms and improve neuron survival [119]. Alternatively, non-pulsatile stimulation of DA production is often used in dopaminergic pathway strategies, which can significantly enhance current treatments [120].

Importantly, Basu Sambuddha and colleagues created a cell line that was expressing SNCA labeled with a nono-Luc luciferase reporter by using the CRISPR-Cas9 strategy. A linear rise in luminous activity was observed as cell numbers were increased from 2500 to 50,000 copies. Their finding revealed that SNCA transcription is monitored endogenously, suggesting that it could be used as a drug testing technique for future PD therapies [121].

The limitations of CRISPR-Cas9 systems include Cas9 delivery efficiency into cells or tissue, off-target effects, and ethical concerns about using CRISPR technology in humans [122]. Although these therapies seem to be quite interesting and effective, more research is required to determine that they can be utilized safely.

## 6. Disease Modeling and Genetic Screening

Targeted gene modification using CRISPR/Cas9 technology is a powerful method for studying gene function and precisely manipulating cellular behavior and function. Moreover, this tool enables genetic engineering at the organism level to create animal models to better understand the etiology and molecular mechanisms of various diseases that can be applied for therapeutic strategies [123].

Animal models are crucial in screening novel pharmacological agents and developing new PD treatment strategies [124]. The relevance of a disease model in terms of predictive validity and construct validity must all be considered when choosing a disease model [125]. The selection is crucial because the research’s translatability depends on appropriate animal models to mimic the human condition or pathology [126]. Moreover, the development of animal models focuses on one or more primary mechanisms associated with PD, such as mitochondrial dysfunction, oxidative stress, and cell neuroprotection [127,128].

As PD is a complex disease with an extensive range of symptoms and development rates, it needs a wide range of animal models to investigate its various features and biological characteristics. Worms, flies, mammals (rodents, primates, cats, minipig, and dogs), and other animal species (such as drosophila and zebrafish) have been used in PD research [129]. Worms and flies are beneficial for investigating individual pathogenic pathways; however, rodents and non-human primates are being studied more closely to understand human diseases better. Rodents are the most common animal species used in PD research because they have many genetic similarities to human anatomy, are easy to handle, do not require a unique breeding setup, and are moderately smaller [130,131].

Numerous genetic studies have provided a better understanding of the potential etiology of PD with family history-specific mutations in the SNCA, PARK2, LRRK2, PINK1, and DJ-1 genes [132]. SNCA is linked to α-synuclein expression, and it is the most important predictor of sporadic PD [133]. Chen et al. used isogenic human induced pluripotent stem cell-derived neurons from PD patients with A53T and SNCA triplication, autosomal dominant mutations, and their associated corrected cell lines by genome editing to examine the molecular role of SNCA in the nucleus. For the first time, it has been postulated that α-synuclein interacts with Ras related nuclear protein and operates properly in nucleocytoplasmic transport constituents while also exerting its pathogenic effect by sequestering the Ras-related nuclear protein. It is concluded that a common pathomechanistic driver of neurodegenerative disorder is mainly the result of defects in the nucleocytoplasmic transport constituents [134]. This mechanism was further validated in CRISPR-edited iPSCs [135]. Another study found that the distal regulatory SNP locus rs12411216 could influence glucocerebrosidase gene expression and enzymatic activity, as well as increase α-synuclein aggregation, which indicates its importance in the pathophysiological development of PD. This study shows the interaction between glucocerebrosidase mutation and α-synuclein aggregation, as well as the fact that the rs12411216 SNP is a causal variable that might be used as a de novo biomarker for mild PD cognitive impairment prognosis [136]. Another study found that SNCA-depleted cell lines are resistant to Lewy pathology [137]. CRISPR-Cas9 is a valuable technique that helps researchers in PD investigations establish isogenic cell lines for PD modeling because genome editing tools could contribute to studying PD phenotypes. Isogenic pairs of cell lines are those which vary only by a single genetic alteration, and are useful tools for figuring out how genes work. However, it is laborious, time-consuming, and in some cases impossible to create a pair of mammalian cells [138]. Arias-Fuenzalida et al. used fluorescence-activated cell sorting-assisted CRISPR-Cas9 editing to develop the set of isogenic lines with human SNCA mutants and the relevant PD phenotype [139].

Because of their roles in mitochondrial function control, PINK, P13, and PARKIN are also emphasized as potential therapeutic targets. Mutations in Parkin and PINK1 homologs cause mitochondrial dysfunction in flies, resulting in DA neuron loss, mitochondrial augmentation and decomposition, muscle degeneration, and a limited lifespan [140,141]. Parkin and PINK1 knockout mice, on the other hand, were unable to reproduce the PD-related symptoms seen in human patients [142]. Furthermore, in neuronal cultures and in vivo, mitophagy pathways that do not depend on Parkin and PINK1 have been discovered [143,144]. Several studies have demonstrated PINK1 knockout animal models [145,146]. In CRISPR-edited PINK1 knockout rhesus monkeys, Yang et al. found a substantial number of neuronal deletions, but not in PINK1 defective mice or pigs. This discrepancy can be explained by PINK1 activity and expression in primates [147]. PINK1 is hypothesized to have a wide range of properties. The phenotypic intensity and difficulty caused by PINK1 deletion may vary depending on the number and type of single-gene mutations. Moreover, variable degrees of PINK1 deletion can be caused by a mosaic of CRISPR-Cas9-mediated mutations [147]. Several attempts have been made to use CRISPR-Cas9 technology to explore and cure PD-related diseases linked with Parkin/PINK1-dependent mitophagy dysfunction. However, in animal models of PD, knocking out PINK1 and Parkin did not replicate the PD-related behavioral patterns and pathological abnormalities reported in patients [146,147]. PARKIN mutations have been investigated in iPSC lines to see how they affect PD-related protein expression [148]. These findings show that the pathology of PD is very complicated, and it is dependent on the proper operation of numerous different processes.

Several researchers have proposed creating an LRRK2-related PD stem cell model [149]. Mutations in the LRRK2, associated with its enhanced aberrant activity and resulting in DNs toxicity, are the most common genetic cause of sporadic and familial PD. The p.G2019S mutation is the most frequent in the LRRK2 gene, and the G2019S LRRK2 mutant is suggested to directly interact with and phosphorylate α-synuclein, resulting in its aggregation and cell death. LRRK2 is a notable component of LBs found in human PD brain samples. In patient-derived human induced pluripotent stem cells (hiPSCs), Qing et al. applied the CRISPR-Cas9 and piggyBac technologies to generate the LRRK2-G2019S isogenic hiPS cell line, which recapitulated the cellular phenotypes observed in DNs from patients with the LRRK2-G2019S mutation with the decrease of the tyrosine hydroxylase (TH) positive neurons [150]. Another LRRK2 iPSC model created using the TALEN method could also be used as a reference [151].

Another protein, DJ-1, is related to reactive oxygen species (ROS) formation, oxidative stress, autophagy regulation, and mitochondrial function [152,153]. Autosomal-recessive early-onset PD has been linked to mutations in the DJ-1 gene (PARK7) [154]. Hao et al. revealed that knocking out DJ-1 in mice and drosophila resulted in age-dependent mitochondrial dysfunction. DJ-1 knockout flies, like Parkin and PINK1 mutants, have male sterility, restricted climbing abilities, and a short lifespan [154]. Parkin and PINK1 mutants have the same phenotypes as DJ-1 knockouts, such as male sterility, reduced climbing ability, and limited lifespan [155]. Although Parkin/PINK1 and DJ-1 may be involved in two distinct pathways that are both important for mitochondrial activity, their exact interactions remain uncertain [154]. It has also been found that DJ-1 can decrease α-synuclein aggregation and toxicity [156]. DJ-1 has been shown to interact directly with monomeric α-synuclein in vitro and in vivo cells, reducing its dimerization. On the other hand, DJ-1 mutants were unable to prevent α-synuclein dimerization and so lost their protective qualities [157].

Pigs have several good anatomies, physiology, and genetic qualities that make them better models for human disorders, particularly NDs, because their brain convolutions are like those of the human neocortex. Wang and colleagues, by co-injecting Cas9 mRNA with multiplexing single guide RNAs (sgRNAs) into in vivo derived pronuclear embryos, simultaneously targeted three unique genetic loci, parkin/DJ-1/PINK1, to create a human PD pig model in Bama miniature pigs. The findings show that the CRISPR-Cas9 system’s simplicity, efficiency, and power may be used to modify numerous genes in pigs and produce medically beneficial results [158]. Zhou et al. used Cas9/sgRNAs to effectively direct gene editing in pig fetal fibroblasts and then used mutant cell colonies as donors to make homozygous gene-targeted pigs with a single somatic cell nuclear transfer (SCNT) round. The combined CRISPR-Cas9-SCNT system allowed for the creation of single- or double-gene targeted pigs without mosaic mutation or obvious off-target consequences. This method offers the development of genetically modified pigs or other large animals in a more efficient, quick, and cost-effective manner [146].

Monkey research is crucial in the pre-clinical development of therapies and is effective for a psychological examination of more complicated behaviors because monkeys are more closely related to humans [159]. Chen et al. validated that microinjection of two truncated sgRNAs and Cas9-D10A mRNA into embryos of one-cell stage cynomolgus monkey resulted in effective gene alterations, enabling one-step creation of PINK1 mutant monkeys, and did not cause observable indels in the top 13 possible off-target sites. The result shows that paired Cas9 appears to be an effective and precise method for establishing human disease models in non-human primates [160]. Hao Li et al. directly coedit DJ-1 and PINK1 genes in the substantia nigras of middle and old aged monkeys using the AAV9-delivered CRISPR-Cas9 system. It has been demonstrated that the middle-aged monkeys acquired PD signs but to a limited extent compared to the older ones. This indicates that aging plays a role in the progression of PD. However, it is still unclear how this model resembles the developing process of early-onset familial PD [161].

## 7. Delivery of CRISPR-Cas

The CRISPR-Cas9 gene-editing technology has completely transformed the research field. Continuous efforts in developing this science have provided excellent in vivo, in vitro, and ex vivo gene editing using diverse delivery strategies [162]. Importantly, this also has emerged as a powerful tool to manipulate the genome for therapeutic purposes to treat various genetic disorders such as thalassemia, tyrosinemia, or cancers [163]. In order to successfully deliver the CRISPR-Cas9 genome editing system, both physical techniques and delivery vectors are usually used. mRNA or plasmid expressing nucleases can be delivered to target tissue or cells using delivery vehicles like viral and non-viral vectors. Alternatively, for the delivery of nuclease into cells, physical means such as laser, physical energy, ballistic delivery, microinjection, or electroporation can be used [164]. Because of the drawbacks of viral vectors, such as immunogenicity, carcinogenesis, and low encapsulating capacity, non-viral vectors are preferred [165,166,167]. Only a few non-viral vectors for gene therapy have entered clinical trials due to their poor in vivo delivery effectiveness [168]. We summarized techniques for delivering CRISPR-Cas9 systems into cells in this context.

### 7.1. Viral Vectors

Infection and replication are the two processes through which viruses deliver their information. A virus can detect and penetrate a specific cell during the infection stage, and the viral genome is released into the cytoplasm in case of cytoplasm or nucleus in case of DNA for its replication. Replicated virions leave the cells once the viral genome has been replicated in the cells. In nearby cells, the infection stage begins again, and the infection–replication cycle continues [169]. Gene therapy can be accomplished by genome editing when the virus transports the delivery materials to the targeted cells. Virus vectors were the first vehicle to efficiently deliver CRISPR genome editing components. Retroviruses, adenoviruses, lentiviruses, and adeno-associated viruses are the most successful viral vectors [170]. These vehicles can carry sequences ranging in length from 4.5 to 5 kb, usually including sgRNA as well as a short regulatory component with promoter and polyadenylation sequence information, depending on the virus [171].

The Adenoviridae family of adenoviruses (AVs) was first discovered in human adenoid cells in 1953. AV-mediated delivery can be used in both in vitro and in vivo conditions in general [172]. Many cell lines were effectively delivered with RNA-guided nuclease [173]. Adenoviral vectors (AdVs) are frequently used in clinical trials to deliver genes. AdVs can target both nondividing and dividing cells, but they do not incorporate into the genomes of their hosts [174]. The researchers examined second-generation fiber-modified AdVs that expressed the Cas9 element or gRNA component transduced into a safe harbor gene AAVS1 or a recombinant allele that can be produced in high titers [173]. A significant disadvantage of AdV-mediated delivery is that it can activate a high number of innate immune responses, leading to AdV removal and tissue inflammation [174]. The production of AdVs takes time [174], limiting the strategy’s use and efficacy.

Adeno-associated viral vectors (AAVs) have distinct advantages over other viral vectors, like better delivering capabilities and non-pathogenic characteristics, which has led to increased use in gene editing and CRISPR application [175]. Even though inflammatory reactions have been described as a potential problem in AAVs, they remain a preferred choice [176]. AAV’s safety and biocompatibility were confirmed in many clinical trials, leading to the FDA’s approval [177]. The unique characteristics of AAV, including replication failure, lack of genomic integration, and human minimum immunogenicity, have aroused interest in its use as a vector, especially in vivo [178]. After transduction, the AAV genome is retained episomally in the nucleus and then diluted through the process of cell division. Hence, in vitro AAV gene delivery via the episomal vector is a safe transient gene expression approach [176]. Alternative AAV variants boost viral capacity and specificity and have been tested successfully. Modifications to the capsid proteins are the most common. Concatemers of AAVs have a long life cycle and can be utilized to express transgenes for a long time [177]. Peptides introduced to the AAV genome’s VP3 region cause vector re-targeting, which improves specific-organ transduction. As a result, changes to the VP2 region can affect the vector’s viral delivery efficacy and transduction capability [179]. Capsid engineering is a potential method for meeting the unique requirements of gene editing applications. The virus may successfully target the central nervous system and pass across the blood-brain barrier (BBB) due to its inherent directivity [180]. Because of the BBB’s limited permeability, transduction in the CNS can fail, making this change necessary for CNS gene editing [181]. BBB is a filter that can capture particles bigger than 400 Da, making it difficult for virus vectors to pass through [182]. Another limitation of AAVs is their limited gene targeting efficiency. Only 0.1% to 1% of the total number of cells perform specific homologous recombination under suitable conditions [183]. ZFNs are currently used in only AAV-based gene editing trials that are registered on ClinicalTrials.gov to integrate precise copies of genes into the genomes of individuals with mucopolysaccharidosis types I and II [184] or hemophilia B [185]. Clinical experiments using AAV-based CRISPR-Cas9 gene editing are expected to begin soon, as AAV-based delivery is likely to become more popular. Furthermore, AVV vector-based treatments can be costly, making them unsuitable for several CRISPR and gene delivery applications [186]. Lentivirus (LVS), another CRISPR-Cas9 vector, has larger cloning efficiency (8KB) than the AVV vector, allowing sgRNA and Cas9 to be cloned into a single LV vector. LV synthesis is also less time-consuming than AAV production. In many cell types, both dividing and nondividing, the LV transduction mechanism is extremely efficient [187]. The LV vector is an ideal choice for in vitro and in vivo delivery because of these benefits [187]. Nevertheless, the most challenging part of LV systems is random integration into host cell genomes. The incorporation of LVs into oncogenes may result in their activation and tumorigenesis [188].

### 7.2. Non-Viral Vectors

For in vitro and ex vivo CRISPR systems, physical delivery is a common approach [189]. This procedure significantly enhanced the amount of genetic material that is easily accessible. Microinjection, electroporation, and hydrodynamic delivery are typical physical delivery techniques [190]. Physical administration in zygotes has been employed to create ex vivo transgenic animals, although these approaches were not intended for in vivo studies due to structural cellular damage [191]. A physical approach is a microinjection, which employs a glass micropipette to administer RNP precisely into living cells. It bypasses the molecular weight barrier by allowing accurate control of the injectable Cas9-sgRNA complex [192].

Injection of RNP into embryos of various organisms has been successful so far, including mice [193], zebrafish [194], rabbit [195], reef-building corals [196], and axolotl [197], olive fruit fly [198], and spider mite [199]. Contrary to this, embryo microinjection may result in inevitable cell damage, and it necessitates highly skilled manipulation and sophisticated tools, both of which are difficult to implement in non-specialist facilities. Furthermore, because their eggs are fragile or non-oviparous, some species are sensitive to embryonic microinjection [200].

Furthermore, electroporation uses an electrical pulse to disrupt the phospholipid bilayer of cell membranes, resulting in temporary nanopores through which biomacromolecules such as proteins, nucleic acids, and RNPs can pass [201]. Due to the successful delivery of cargoes into a broad range of cells, electroporation is commonly used for ex vivo and in vivo gene editing. This is superior to conventional transfection procedures, which are usually confined to difficult-to-transfect cell types like primary cells. Ex vivo gene editing through electroporation has helped in the development of stem cells for the treatment of hematologic malignancies [202].

Biolistics, which stands for “biological ballistics”, is a direct physical approach for delivering biomacromolecules primarily into plant cells. The biomolecules were encapsulated onto tungsten or gold microparticles, which were then accelerated to incredible velocities with high-voltage electronic discharges, chemical explosions, helium shock, or pressurized gas [203]. Magneto-electric nanoparticles (MENPs) have been shown to be an effective magnetically guided approach for delivering CRISPR-Cas9/gRNA nanoparticles across the BBB without interfering with its cellular junction [204]. As a result, the attached biomolecules might be shot through cell walls and membranes into target cells. Using this biolistic method, pre-assembled RNPs were delivered into maize embryo cells, and as a result, there was a higher frequency of maize with changed alleles and better gene mutations (Table 2) [205].

LNPs (lipid-based nanoparticles) are a non-viral chemical approach for delivering nucleic acids. For nucleic acid delivery, lipid-based nanoparticles (LNPs) are often used [291]. As LNPs address BBB permeation well, they are widely developed for CNS-targeted therapy [292]. Liposomes are perfectly circular lipid bilayer entities that form in an aqueous solution. Cell membranes and nucleic acid repel each other because they are both negatively charged, preventing nucleic acids from entering cells. The complexes are more accessible to fuse across cell membranes and enter cells when encapsulated in positively charged liposomes [291]. Cationic lipids, which readily form a complex with negatively charged nucleotide sequences and encourage RNA and DNA loading, can be used to make the carriers [293]. As a result, lipid carriers are ideal for delivering plasmids carrying CRISPR editing systems’ sgRNA and endonuclease sequences. Furthermore, lipid-based vehicles effectively transport RNPs or even nucleic acids and proteins in combination [190].

Polymer carriers for gene and oligonucleotide drugs are also used. The development of cyclodextrin conjugates with the starburst polyamidoamine (PAMAM) dendrimer (CDEs) for passive and active targeting genes have been progressing. Importantly, CDE has been demonstrated to be a useful Cas9-RNA ribonucleoprotein (Cas9 RNP) carrier for gene editing in the neuron and brain [294].

The main challenge in developing a brain-directed therapy is getting it past the BBB, which isolates and protects neural tissue while also managing the entry of molecules and thus obstructs delivery. In the Comprehensive Medical Chemistry database, approximately 7000 drugs have been evaluated, with only 5% of them being able to cross the BBB and enter the CNS [295]. Various strategies have been demonstrated for efficiently delivering components to neural tissue [296]. In 2014, Agustín-Pavón and his research group demonstrated several invasive and less invasive procedures to successfully deliver components into the neural tissue. The invasive methods involved direct injection during stereotactic surgery into the ventricles or the parenchyma of the brain. Furthermore, laser irradiation, microbubbles with ultrasound activation, and the entry of hyperosmotic solutions are also involved in more invasive methods [296]. Intranasal access with nanoparticle (NP)-assisted drug delivery across the BBB is a method that plays a significant role in reducing invasive impact due to the application of solid colloidal NPs with sizes ranging from 1–1000 nm (polymers, lipids, magnetic liposomes) [297]. In addition, NPs modification with cationic stabilizers or non-ionic surfactants enabled successful BBB passage with subsequent cellular labeling [298]. Another noninvasive procedure includes exosomes, cell-penetrating peptides (CPPs), often called protein transduction domains (PTDs) or “Trojan horse” peptides, which as a group of diverse peptides with a size ranging from 5–30 amino acids (4–24 nm) have the efficiency in penetrating through cellular plasma membrane [299]. The methods mentioned above can be used as a carrier to enhance the efficiency of CRISPR systems by taking advantage of crossing the BBB, therefore possibilities of the target site editing could be improved.

The intranasal route of administration allows therapeutic biomolecules to be delivered directly to the CNS, bypassing the BBB without needing surgery. The mechanisms that support nose-to-brain transport have previously been discussed [300,301]. Peptides, proteins, siRNAs, viral gene vectors, non-viral gene vectors, and even cells have been shown to transport from the nose to the brain successfully. Peptides and proteins have been the most extensively researched of these. Insulin, melanocortin, and arginine vasopressin were administered intranasally to healthy humans in one of the first studies [302]. Previously, the efficacy of noninvasive intranasal delivery of siRNA by applying the cationic linear polyethyleneimine to the brain of the adult mouse brain to achieve HIV attenuation has been demonstrated [303].

Exosomes are naturally occurring membrane-bound vesicles with high biocompatibility and minimal immunogenicity. They can transport proteins, plasmids, miRNAs, and siRNAs, among other biomolecules, that could be used as cell-free therapeutics [304]. Therefore, engineered exosomes have been used to deliver drugs to specific locations [305]. Previous studies suggest the possibility of targeting exosomes for small molecule and miRNA delivery in cartilage regeneration [306]. Exosomes can encapsulate the components of Cas9 and gRNA proteins, allowing the CRISPR-Cas9 system to transfer gene editing activity into cells. Bioengineered Vero and CHO cells can create exosomes containing functional gRNA and Cas9 proteins. In a unilateral 6-OHDA rat model, the therapeutic impact of extracellular vesicles (EVs) produced from human exfoliated deciduous tooth stem cells (SHED) was recently determined for the first time. In the striatum and substantia nigra, they improved gait and normalized TH expression [307]. The accumulation of antioxidant proteins thioredoxin (TXN), Cu/Zn peroxiredoxin-6 (PRDX6), and superoxide dismutase 1 (SOD1) in EVs may reduce the sensitivity of DNs to 6-OHDA, inducing oxidative stress according to the previous studies [308]. This could be a promising non-viral therapeutic alternative for PD that has minimal invasive adverse effects. Although there is increasing interest in non-invasive gene editing methods that bypass BBB, more research is still needed on its safety and effectiveness. Exosomes can indeed transport Cas9 proteins and gRNA to targeted cells, enabling the CRISPR-Cas9 system to function [239]. Because of the Cas9 and gRNA’s quick elimination and their outstanding biocompatibility, Cas9 and gRNA delivery decrease the threat of off-target activities and genomic integration. Therefore, exosomes may be the safest and most efficient way to deliver the CRISPR-Cas9 system.

## 8. Challenges and Future Perspectives

PD is a collection of movement disorders characterized by abnormal and undesirable involuntary movements. Selective neuronal loss in parts of the brain involved in fine-tuning activity is the major cause of the patients’ different motor symptoms. However, this knowledge of conventional therapies cannot prevent, reverse, or slow the progression of PD. Current treatments aim to keep motor symptoms in check, but they are ineffective. As a result, constant and concerted attempts are underway to establish innovative disease treatment strategies. The failure rate of small molecule development for the treatment of NDs is high. Novel approaches such as gene therapy, cell transplantation therapy, and immunotherapy are still being extensively investigated in animal models. While there are a lot of lingering questions about these new therapies, such as their safety and efficacy, there is a strong likelihood that we will see them in clinics for PD treatment soon. As a new genome-editing technique, CRISPR improves biomedical research and treatment strategies for PD [309]. This system allows for genome alterations, including the deletion of long nucleotide sequences, homologous recombination, insertion/deletion point mutations, and transcription manipulation of specific genetic elements. Due to its functional nature and resistance to epigenetic changes, CRISPR-Cas9 is now the most helpful technique for genome editing in human disease modeling in vivo and in vitro [310]. However, as we discussed above, the main limitation of effective gene therapy for PD is still the poor understanding of its pathogenic mechanisms. Nevertheless, with an increasing number of studies focusing on discovering the molecular mechanisms and developing gene therapies for this disease, the CRISPR-Cas9 technology offers significant potential to be applied both to improve our understanding of PD and to achieve successful future treatments targeting the proper genes.

Despite its numerous advantages, multiple aspects of the practical application of CRISPR-Cas9 must be optimized to maximize its functionality. DSB that can cause unexpected DNA changes or even lethality of some cells has been demonstrated. Therefore, non-DSB and template-free genome editing types such as base editing (BE) or prime editing (PE) are developing [311]. Cytosine and adenine base editing in mouse brains has been reported to effectively correct a mutation causing neurodegenerative ataxia, as slowing down neurodegeneration and increasing the animals’ lifespan has been demonstrated [312]. In the G93A-SOD1 mouse model of ALS, treatment with CRISPR base cytidine editors reduced the rate of muscle atrophy and muscle denervation, improving neuromuscular function [313]. The high frequency of off-target genome modifications by CRISPR-Cas9 is a major challenge. Single and double-base mismatches may also be tolerated to variable degrees at the gRNA-DNA interface, resulting in undesired mutagenesis [314]. Moreover, gRNA and Cas9-encoding plasmids show continuous gRNA-Cas9 complex formation, which could lead to an accumulation of off-target mutagenesis. To prevent possible off-target regions in the genome, cloud-based techniques can now be used to build unique target sequences. Another option is to use recombinant Cas9 proteins, which produce mutations at on-target sites as soon as they are introduced into cells and are promptly eliminated by cellular protease machinery, reducing off-target effects [315]. In addition, a relatively low Cas9 concentration can reduce off-target effects while sacrificing on-target efficiency [316]. By employing precise gRNAs and appropriate Cas9 concentrations, CRISPR-Cas9-mediated gene targeting should become more specific and decrease off-target effects. Because of the technology’s novelty, the long-term effects of undesirable mutagenesis induced by CRISPR-Cas9 in humans are difficult to predict, as evidenced by the expert debate over the promising long-term medical advantages and the negative side effects encompassing the first CRISPR-Cas9-edited baby born in China in 2018 [317]. Before this technology may be employed in therapeutic settings, ethical and safety considerations must be resolved. The inconsistency of CRISPR-Cas9-based treatments’ delivery to target cells is also major issue.

Another problem with CRISPR-Cas9 is mosaic mutations, which can be caused by prolonged Cas9 expression after cell division or by a slow rate of Cas9 nuclease cleavage. Conversely, non-homozygous recombination activities and differential DNA repair can alter genetic mutation levels and mosaicism in polarized embryonic cells and zygotes. Some scholars have also tried to transport Cas9 proteins directly into cells with relatively high effectiveness, however, mosaic mutations persist [260]. Cas9 nuclease expression in zygotes can be closely controlled at the transcriptional and translational levels, potentially reducing mosaic mutations.

The reduced rate of homologous recombination is another dilemma for CRISPR-Cas9. HDR occurs in the synthesis (S) and pre-mitotic (G2) phases [318], whereas NHEJ normally occurs during the mitotic (M) stages and growth 1 (G1) [319]. The HDR rate is low, despite the high efficacy of CRISPR-Cas9-mediated indel mutations via NHEJ. By suppressing NHEJ critical molecules, CRISPR-Cas9 has been shown to boost HDR rates [320].

Another critical issue is the emergence of immunological reactions when CRISPR is used, and a person’s body fights against gene therapy. The consequences of anti-Cas9 responses were demonstrated using a sample of 34 human blood cells in a recent study. Antibodies to SaCas9 and SpCas9 were found in 79% and 65% of the samples, respectively. This issue will have to be addressed in clinical applications to properly utilize CRISPR’s promise to combat genetic disease [79]. Furthermore, several limitations exist for the effective delivery of the CRISPR-Cas9 component through lipid nanoparticles. The first step is to consider both external and internal obstacles. After passing through the cell’s surface, the nanoparticle is usually wrapped in an endosome. Cells can rapidly guide the encapsulated contents through the lysosomal pathway, resulting in the degradation of all lysosomal contents. Therefore, the cargo must be able to escape the endosome. In addition, if the Cas9: sgRNA complex can escape the endosome, it must then translocate to the nucleus, which can be a point of failure. As a result, high efficacies when delivering CRISPR-Cas9 components via lipid nanoparticles are rare [190]. Recently, nanostructured materials (both organic and inorganic) have emerged as valuable agents for effectively internalizing cells and thereby increasing the efficacy of gene-editing methods [321].

Another disadvantage of CRISPR utilization is the lack of an efficient delivery strategy for accessing the CNS framework. Suitable delivery vehicles may be able to help overcome some of the present issues with CRISPR gene editing applications [190]. Lentivirus, AAV, electroporation, microinjection, and liposomal or nanoparticles are all standard delivery methods, and some of them work for CRISPR-Cas9-based therapeutic delivery in the CNS [322]. Furthermore, to overcome this limitation, a neuron-preferring chimeric AAV-based CRISPR-Cas9 approach for inducing brain-specific gene deletion has been developed [323].

However, there are numerous hurdles connected with CRISPR-Cas9 delivery, and we expect that as materials research and nanotechnology progress, more robust delivery techniques will be developed to overcome these obstacles, allowing the clinical application of “magic scissors” to move forward.

## 9. Summary

New therapeutic approaches for PD treatment have recently been developed due to several challenges and drawbacks with present medications in terms of adverse effects, efficacy, and cost. Cell transplantation therapy, immunotherapy, and gene therapy are examples of innovative techniques that are still being developed and evaluated in animal models. However, there remain many unanswered questions about these novel technologies, such as their safety and efficacy, but there is still a significant chance that we will see the use of these strategies in the clinic for PD therapy in the near future. The treatment of hereditary diseases with gene therapy is a source of great promise for scientists. In vitro and in vivo, gene editing methods can solve the problem of gene defects by permanently altering a genomic sequence of interest through insertion, deletion, disruption, and correction. Additionally, sufficient study is required to enhance the procedure and validate the standard efficacy in the animal model. CRISPR could be employed in a variety of clinical settings, including adult stem cells, ESCs, and iPSCs. It would also be essential to investigate whether similar gene-correction technologies could be used to repair mutations in PD diseases. Though CRISPR-Cas technology has a good prospect for long-term gene editing, it is still difficult to bring it into clinics because of immune system activation, off-targeting, and poor in vivo delivery.

## Figures and Tables

**Table 1:** Genes implicated in the development of PD, their loci, proteins, functions, phenotypes, and neuropathology.

Genes	Gene Locus	Alternative Names of the Gene	Proteins	Gene Function	Results of Gene Mutation	Onset of PD	
PRKN	6q26	PARK2	Parkin	Parkin is a 465-amino-acid cytosolic E3 ubiquitin ligase that participates in proteasome-mediated protein degradation. It damages misfolded and overproduced proteins, as well as ubiquitin.	The absence of LB, dopaminergic neuron apoptosis in the SN, and neurofibrillary	Early	[80,81,82,83,84,85]
SNCA	4q22.1	PARK 1/PARK 4	α-synuclein	The SNCA gene produces a protein called -synuclein, widely distributed in neurons. Its function is unknown; however, it may be involved in regulating vesicular and dopamine neurotransmission.	The broad presence of LB throughout the brain and cerebral cortex, as well as neuronal destruction in the LC and SN	Early	[86,87,88]
PINK1	1p36.12	PARK6	PTEN induced putative kinase 1	The mitochondrial function of this protein is to protect the mitochondria from the damaging effects of cellular oxidative stress.	The occurrence of LB in the reticular nuclei of the brainstem and neuronal loss in the SN pars compacta	Early	[89,90,91]
RAB39B	Xq28	None	RAB proteins, like RAB39B	These are members of the GTPase family. RAB39B controls the movement of vesicles between membrane compartments.	Extensive dopaminergic neuron loss in SN and classical LB disorder	X-linked early-onset	[92,93,94]
D-J1	1p36.23	PARK7	DJ-1	Several tissue and organs, including the brain, contain the DJ-1 protein. This protein acts as a chaperone molecule and prevents cells from oxidative stress. DJ-1 assists in the refolding of damaged proteins as well as the assembly of specific proteins into the right three-dimensional shape.	LB pathology	Early	[95,96,97,98]
LRRK2	12q12	PARK8	Leucine-rich repeat kinase 2	The protein Roco family includes the component of the gene LRRK2. It is involved in cytoskeletal dynamics, autophagy, and vesicular transport.	Heterogeneous: degeneration of neurons in the SN and occurrence of LB in the brain; specific cases: Neurofibrillary tangle pathology, lack of LB, and neural nigral degeneration	Late	[99,100,101]

PD, Parkinson’s disease; SNCA, Synuclein alpha; SN, substantia nigra; LB, Lewy body; LC, locus coeruleus; LRRK2, leucine-rich repeat kinase 2; PINK1, PTEN-induce kinase 1.

**Table 2:** Summary of various delivery systems for CRISPR-Cas9.

Delivery System	Cas9 Delivery Format	Benefits	Limitations	References
**Viral Approaches**				
Adenoviral associated virus (AAV)	DNA	Applicable in vivo, safe, non-integrating, low immunogenicity, nucleic acid size < 5 kb, high infection efficiency	Limited cloning capacity, production difficulty.	[206,207,208,209,210,211,212,213,214,215,216,217]
AV	DNA	Applicable in vivo, nucleic acid size—8 kb, non-integrating	Immune response	[218,219,220,221,222,223,224]
Lentiviral Virus	DNA	Applicable ex vivo and in vitro; high infection efficiency, nucleic acid size 10–18 kb, persistent gene expression, efficient delivery, high capability for cloning	Capability for insertional mutagenesis, random integration, transgene silencing	[209,225,226,227,228,229,230,231,232,233]
EV	Protein	Applicable in vivo, in vitro, and ex vivo, non-integrating, multiplexable, transient exposure	Restricted quantification technique	[234,235,236,237,238,239]
**Non-Viral Approaches**				
Microinjection	DNA, mRNA, or Protein	Applicable in vitro and ex vivo, targeted delivery, precise and reproducible	Laborious, cell damage, need a high level of skills, mostly used in vitro	[191,206,240,241,242,243,244,245,246,247,248,249]
Electroporation	DNA, mRNA, or Protein	Applicable ex vivo and in vitro, accessible, high rate of transfection, targeted delivery	Cell viability problem, generally in vitro only	[226,250,251,252,253,254,255,256,257,258,259,260]
Mechanical cell deformation	Plasmid based CRISPR-Cas9	Relative low number of cell death, efficient delivery	Mostly used in vitro	[261,262,263]
Hydrodynamic injection	DNA, protein, siRNA	Suitable for hepatocyte transfection, feasible, low cost, applicable in small animals (in vivo transfection)	Nonspecific, causing tissue damage, not applicable for large animals	[264,265,266,267,268,269,270,271,272,273]
Lipid nanoparticle	DNA, mRNA, or Protein	Applicable in vitro and in vivo, approved by FDA; safe, easy manipulation, minimal stress to cell, low cost	Cargo degradation in endosomes, significant optimization required, cell tropism	[209,274,275,276,277,278]
Gold nanoparticle	Protein	Applicable in vivo and in vitro, inert, high efficiency, membrane fusion like delivery	Potentially harmful in vivo, at high concentrations nonspecific inflammatory response	[279,280]
Polymer nanoparticles	Plasmid DNA, RNA, and oligonucleotides	Safe and easy preparation	Low delivery efficiency	[281,282,283,284,285,286]
Magneto-electric nanoparticles (MENPs)	sgRNA	BBB permeability, non-invasive, controlled release	Magnetically guided	[287]
Cell-penetrating peptide (CPP) delivery	Protein	Small size, can deliver intact RNP into a cell	Variable penetrating efficiency, considerable optimization required	[261,288,289,290]

## Data Availability

Not applicable.
